# A comprehensive cardiovascular disease lifestyle treatment controlled trial among high-risk African Americans

**DOI:** 10.4236/ojpm.2013.39071

**Published:** 2013-10-31

**Authors:** Sharon K. Davis, Rakale Quarells, Gary H. Gibbons

**Affiliations:** 1 National Institutes of Health, National Human Genome Research Institute, Bethesda, USA; 2 Morehouse School of Medicine, Social Epidemiology Research Center, Cardiovascular Research Institute, Atlanta, USA; 3 National Institutes of Health, National Heart, Lung, and Blood Institute, Bethesda, USA

**Keywords:** African American, Cardiovascular Disease Risk Factors, Lifestyle Modification

## Abstract

**Background:**

The purpose of this study was to assess the effects of a comprehensive lifestyle intervention on modifiable cardiovascular risk factors among high-risk African Americans.

**Methods:**

The study included a randomized treatment/controlled intervention trial among 136 African Americans residing in Atlanta, GA who were overweight and had elevated blood pressure. The treatment group was exposed to 3-months of a multi-component intervention and the control to an abbreviated 6-week intervention after the completion of the treatment group’s intervention. The main outcomes included mean systolic blood pressure (SBP), mean diastolic blood pressure (DBP), mean waist circumference, mean body mass index (BMI), mean number of times exercise per week, mean number of servings of fruits and vegetables per day, and mean level of daily stress. Data were collected at baseline and at 6-month follow-up. Separate linear regressions were used with an established significance level of p < 0.05.

**Results:**

Results revealed significant net improvement in treatment group when compared to controls in waist circumference, BMI, times weekly exercise, servings of fruit and vegetables per day (p < 0.001, 0.04, 0.02, 0.002, respectively). Diastolic blood pressure also significantly improved within the treatment group for overall hypertensives from baseline to 6-month follow-up (90.9 mmHg to 83.1 mmHg, p = 0.002).

**Conclusion:**

These results show that a comprehensive lifestyle intervention can improve cardiovascular risk factor profile among high risk African Americans. Caregivers should encourage patients to participate in such programs and public health policymakers should allocate resources to community based health oriented organizations to implement comprehensive lifestyle program.

## 1. BACKGROUND

Cardiovascular disease (CVD) is the leading cause of death and disability among men and women in the United States [1]. Advances in technology, public health campaigns, and public health policies have lead to significant improvement in morbidity and mortality resulting from modifiable behavioral risk factors [2]. Despite such improvements, African American men and women continue to have higher rates of CVD mortality and morbidity due to adverse lifestyle behaviors [2]. Several clinical and community based studies have assessed the effects of comprehensive interventions on improving multiple adverse lifestyle behaviors [3–9]. Studies have shown the benefits of physical activity, a diet high in fruits and vegetables, and stress management as improving CVD lifestyle risk factors [3–10]. Research has consistently showed that African Americans have higher rates of physical inactivity, excess weight, excess fatty diet, and elevated blood pressure [2]. However, published results on the effects of multiple lifestyle interventions among African Americans in a community settings have produced little results. None has been conducted exclusively including an African American study population. The objective of our study was to implement a comprehensive community-based randomized treatment/controlled trial among higher risk African Americans who were overweight and had elevated blood pressure to test the efficacy on improvements in modifiable CVD lifestyle risk factor. Findings contribute to information concerning the effects of lifestyle behavior modification on CVD risk factors in higher-risk African Americans.

## 2. METHODS

The study was entitled the Metro Atlanta Heart Disease Study II (MAHDS II). The design included a randomized trial consisting of a treatment arm and a wait-listed control arm aimed to assess the immediate and 6-month post-intervention effects of a multiple 3-month risk factor reduction model on physiologic change in systolic blood pressure (SBP), diastolic blood pressure, waist circumference, body mass index (BMI), and self-reported change in weekly number of physical activity, daily servings of fruits and vegetables and level of daily stress. African Americans with mild to moderate uncontrolled blood pressure and who were also overweight or obese and ≥31 years of age were eligible to participant. A wait-listed control group approach was used for ethical consideration and has been used in similar studies [3,5]. Recruitment was done throughout metro Atlanta, GA via convenience sampling through radio announcements, flyers, health fairs, etc. Mild to moderate blood pressure was defined based on the most recent criteria by the Joint National Committee on Prevention, Detection, Evaluation, and Treatment of High Blood Pressure at the time of the study as SBP ranging from 130 – 179 mmHg or a DBP ranging from 85 – 100 mmHg [11]. Similarly, overweight and obesity was defined by the most recent criteria established at the time of the study by the National Heart Lung and Blood Obesity Task Force as a BMI ranging from ≥25 – ≥30 [12]. Participants with SBP, DBP and BMI that exceeded the criteria range were excluded.

The study was implemented in January 2008 and concluded in November 2008; it was approved by the Morehouse School of Medicine Institutional Review Board prior to implementation. Informed consent was obtained from participants prior to randomization. Those in the treatment group were assigned to a 3-month intervention comprised of 12 weeks of directed exercise by a certified fitness expert one hour a week, eight weeks of nutrition education taught by a certified licensed dietician once a week for 30 minutes, and eight weeks of stress reduction education taught by a certified psychologist once a week for 30 minutes. The control group was exposed to an abbreviated form of the intervention for six weeks after the end of the 3-month intervention for the treatment group. All activities took place at a community YMCA. Participants attended the YMCA in discrete groups once a week for 2 hours. The first 30 minutes consisted of the nutrition education. The second 30 minutes included stress reduction education. The last 60 minutes included directed physical activity which was also conducted at the YMCA.

The exercise facilitator was trained based on elements in the LEARN manual: lifestyle, exercise, attitudes, relationships, and nutrition [13]. Goal setting of increased moderate intensity exercise to 3 or more times a week for a minimum of 30 minutes was established. The exercise component consisted of a 10 minute warm-up, followed by 40 minutes of moderate aerobics and concluded with a 10 minute cool-down. The licensed dietician also had previous training in the LEARN technique for lifestyle modification and supplemented her curriculum to improve daily servings of fruits and vegetables and reduce consumption of high-fat foods based on work done in the Women’s Health Trial in Minority Populations and the Dietary Approaches to Stop Hypertension (DASH) trial [14,15]. Record keeping of daily food consumption was encouraged for each participant. The diary was reviewed and shared with class each subsequent week. The licensed psychologist has a history of working with African American and is trained in stress modification interventions proved effective in African Americans—including Transcendental Meditation and Cognitive Behavior and Rational Emotive Therapy [16]. Participants were encouraged to establish goals to minimize level of daily negative reactivity to stressful stimuli. Participants also recorded daily exposure to stressors and logged application of learned stress management techniques and corresponding success in lowering negative response. Logs were reviewed in class to discuss response to daily stress.

### 2.1. Main Outcome Measures

The main outcome variables included mean SBP, mean DBP, mean waist circumference, mean BMI, mean weekly number of exercise, mean daily servings of fruits and vegetables, and mean level of daily stress. Power calculations were done on all main outcomes which resulted in an 85% power to detect differences between treatment and control group in a study sample of 130 participants. All measures were obtained by a trained technician who was blinded to participant assignment. Mean SBP and DBP was measured with participant sitting using a random-zero sphygmomanometer with appropriate cuff size taken three times at 5-minute intervals. The resulting measure was based on an average of the three. Waist circumference was based on inches at the largest point around the waist. Body mass index (BMI) was derived based on height to weight ratio to the nearest 1/4 (0.1 kg) for weight and the nearest 1/4 inch (0.6) by a metal rule for height. Weight was measured based on a standard balance scale after removal of shoes. Weekly number of exercise was based on the question, “How many times of week do you exercise that increases your heart rate?” Response was the number of reported times per week. Daily servings of fruits and vegetables were based on the question, “How many times a day do you eat fresh fruits and vegetables?” Response was the reported number of times per day. Both exercise and fruit and vegetable consumption measures were derived from the Behavioral Risk Factor Surveillance Study [17]. Perceived level of daily stress was measured based on the question from a validated instrument, “How would you rank your level of day-to-day stress and worry in your life [18]? Responses were ranked order and classified as very high, high, some, or little/none on a scale from 1 to 4 with 1 being the highest level of stress. We also assessed overall hypertensives, those that were medicated, and those that were not on medication. Means were obtained at baseline, at the end of the 3-month and at 6-month follow-up for the treatment group. Means were obtained on the waited-list control group at baseline (after the completion of the 3-month intervention by the treatment group), and at 6-month follow-up.

### 2.2. Statistical Methods

An intent-to-treat analysis was conducted using separate linear regression models with waist circumference, BMI, weekly times exercise, number of servings of daily fruits and vegetables, and level of daily stress were fit with each as the outcome measures and entered as continuous variables. All analyses were performed using Statistical Analyses Software (SAS) 9.2 [19]. Separate linear models were also fit for overall hypertensives with mean SBP and mean DBP, mean SBP and mean DBP for those medicated, and mean SBP and mean DBP for non-medicated participants. The main exposure variable was the 3-month comprehensive lifestyle intervention. Age, gender, and level of education were included as covariates to control for confounding effects. Separate analyses for treatment and control groups were conducted at baseline and 6-month follow-up to ascertain within group differences. A pairwise comparison between treatment versus control were also conducted at baseline and at 6-month follow-up to ascertain net differences between groups. A two-tailed level of significance was established as *P* ≤ 0.05 with 95% confidence intervals (CI).

## 3. RESULTS

**Figure 1:**
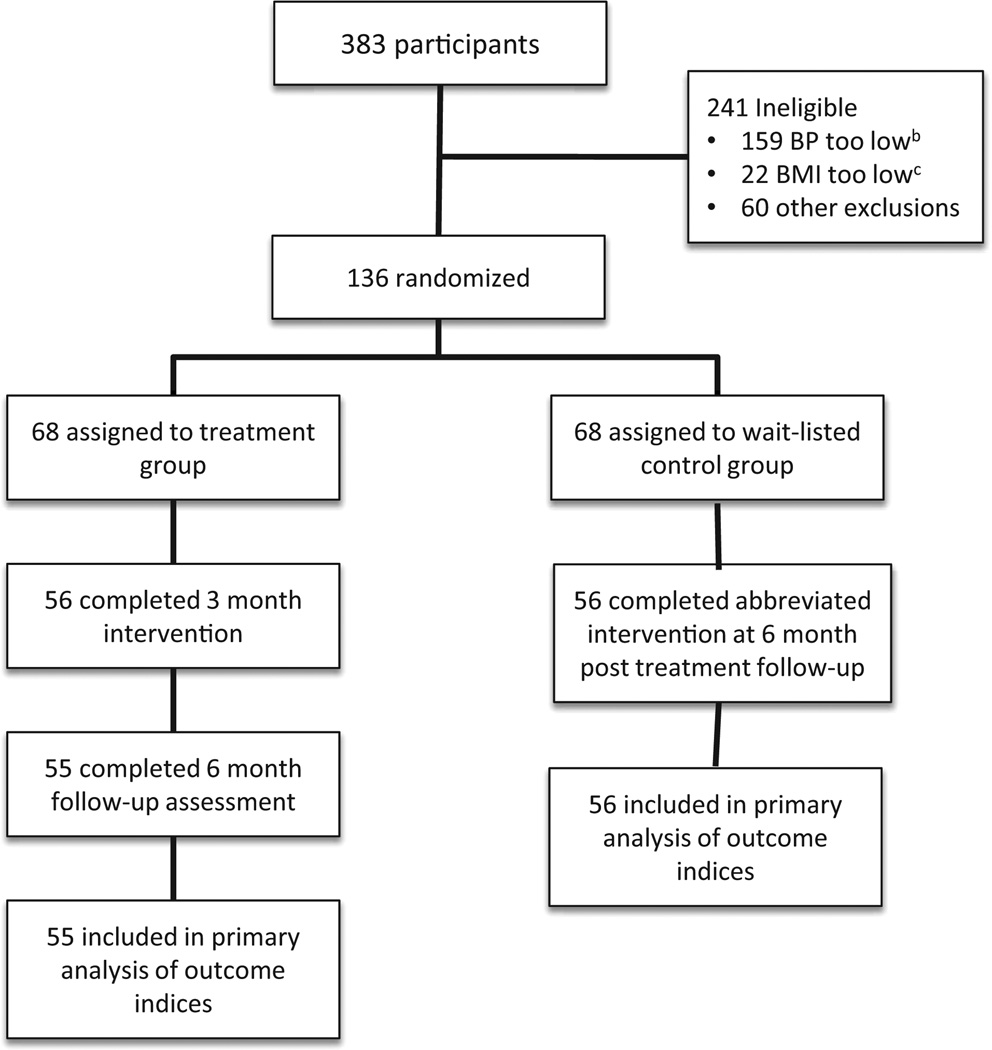
Participant flow in MAHDS^a^ II community-based trial. MAHDS^a^ = metro atlanta heart disease study II; BP^b^ = blood pressure; BMI^c^ = body mass index.

A total of 383 individuals were recruited (Figure 1). Two hundred forty one were excluded because they did not meet the inclusion criteria. The resulting sample included 136 individuals that were randomized to treatment group (n = 68) and control wait-listed group (68) by computer assignment. The groups were randomly divided into 1 of 5 groups of approximately 14 participants. Each of the treatment groups met one evening a week Monday through Thursday and Saturday morning for 3-months. A total of 68 participants started in the treatment group. Fifty six completed the entire 3 month comprehensive intervention for an overall retention rate of 82%. Fifty five completed the 6 month follow-up assessment resulting in a retention rate of approximately 80%. The wait-listed control group was exposed to an abbreviated six week version of the intervention after completion of the treatment group. They were likewise followed-up at 6 months post-intervention. The retention results observed for the control waited-listed group was similar to that of the treatment group. Average attendance for the treatment groups ranged from 80% to 92% for each weekly session

Table 1 reveals the characteristics of the study population. Age is fairly similar between treatment and control groups with a slightly higher age observed in the treatment group. A higher percentage of women were observed in the treatment group (66% versus 61% for control). The remaining characteristics were similar between both groups with the exception of slightly higher number of exercise per week and daily servings of fruits and vegetables among the control group (2.5 versus 1.2 and 2.4 versus 1.6 for treatment, respectively).

Table 2 reveals the results from the outcome measures. Results for SBP and DBP are reported subsequently. Information shows waist circumference, BMI, and level of daily stress did not significantly change within the treatment group from baseline to 6-month post-intervenetion follow-up. However, there was a significant improvement in number of weekly exercise and daily consumption of fruits and vegetables (*p* < 0.001, respectively). There were no significant improvements within the control group from baseline to 6-month follow-up. Net changes at baseline to follow-up between the groups were observed. The control group had higher levels of baseline number of weekly exercise and daily consumption of fruits and vegetables (2.4 and 2.6, respectively). However the net difference in change from baseline to follow-up reveal significant improvements in the treatment group when compared to control group for waist circumference, BMI, weekly number of exercise, and daily servings of daily fruits and vegetables (*P* < 0.001, 0.04, 0.02, 0.002, respectively). There was no significant change difference regarding level of daily stress between the two groups.

**Figure 2:**
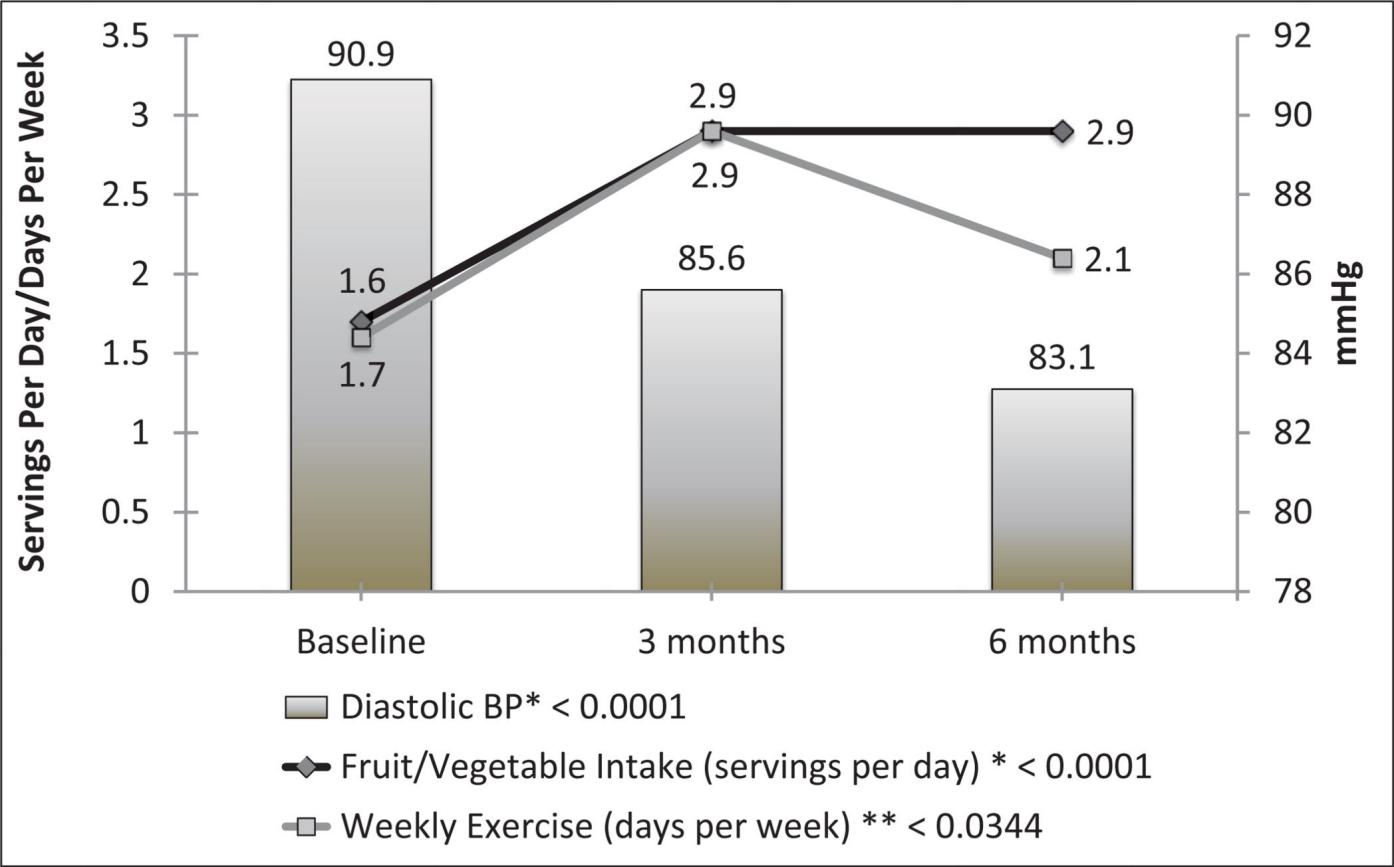
Mean changes in diastolic blood pressure (BP), fruit and vegetable servings, and weekly exercise over time among treatment group.

Table 3 reveals the results of the analyses concerning SBP and DBP. Information shows significant improvement in DBP within the treatment group for overall hypertensives at 6-month follow-up lowering from 90.9 mmHg to 83.1 mmHg (*P* = 0.002). There was marginally significant improvement within medicated and non-medicated participants in the treatment group for DBP from baseline to 6-month follow-up (*P* = 0.07). There were no significant changes among the control groups in the three categories of overall, medicated and non-mediated participants for SBP and DBP. There was also no significant net difference in change between the treatment and control group from baseline to follow-up. Figure 2 illustrates the changes from baseline to end of the 3-month intervention to continual improvement in DBP at 6-month follow-up (*P* < 0.0001). It also shows secular improvements in the treatment group regarding daily servings of fruits and vegetables and days per week of exercise (*P* < 0.0001 and <0.03, respectively).

## 4. DISCUSSION

These results suggest that a multiple community-based life-style intervention can improve behavioral risk factors and blood pressure among higher risk overweight African Americans with elevated blood pressure. The treatment and control groups were not conducted in parallel because intervention exposures occurred at the same site—a local community based YMCA. The treatment group was exposed to a 3-month intervention and the controlled group a 6-week intervention after the completion of the exposure to the treatment group. This approach did not affect the results and is a common approach for such studies [3,5]. Several studies have demonstrated the positive effects of lifestyle interventions among community dwelling individuals [3–10]. Among the first was The Dietary Approaches to Stop Hypertension (DASH) [15]. Previous epidemiologic studies have established the effects of high fat and low fruit and vegetables diet as a risk factor for hypertension [20,21]. The aim of DASH was to assess the effects of a diet rich in fruits and vegetables on reducing SBP and DBP among hypertensives and normotensives when compared to controls following a eight week intervention. Results showed significant improvements in both groups as well as significant improvement among minority participants for both SPB and DBP. It is important to point out that DASH was a control fed diet. Our approach was not, but rather introduced the concept of the health benefits of eating a diet high in fruits and vegetables and reduced high-fat products.

Blumenthal and colleagues were among the first to assess the combined effects of exercise and a weight loss program on blood pressure following twenty-six weeks of intervention [3]. Twenty-three percent of the sample population was African American. Results revealed significant improvements in SBP and DBP when compared to the controls. Outcomes specifically among African Americans were not reported. Oexmann *et al*. conducted a pre/post test analyses and report on the results of an eight week educational session among African Americans in a local Christian community [4]. At baseline 76% of the participants had two or more modifiable risk factors— including overweight and hypertension. The group experienced significant improvements in weight and mean blood pressure following the intervention. Unlike the previous studies, this one did not included specific components for weight or blood pressure reduction. The *D*iet, *E*xercise, and *W*eight Loss Intervention Trial (DEW-IT), however, was among the first to test the effects of a comprehensive lifestyle intervention among overweight hypertensive adults following nine weeks of exposure to the intervention [6]. Components included exposure to directed exercise, the DASH diet, and weight loss education. Participants were randomized to a life-style treatment group or a control group. Results revealed significant improvements in weight reduction and mean SBP and DBP. Sixty eight percent of the sample was African American. However, only aggregate results were reported. Another study published shortly thereafter, reported similar findings [7].

Our study was designed to improve weight, physical activity, consumption of fruits and vegetables, stress, SBP and DBP. We did not observed improvements between the treatment and control groups in waist circumference, BMI, weekly physical activity, and daily servings of fruits and vegetables. We did not observe improvements between the two groups for SBP and DBP—only improvement within the treatment group for DBP. A lack of significant improvement may be because of the similarity in level of SBP and DBP between the treatment and control groups at baseline and 6-month follow-up (Table 3). This was observed in the three categories of hypertensives—overall, medicated, and non-medicated.

Several studies have involved a comprehensive model aimed at ameliorating multiple risk factors [3,6,7]. Few, however, have focused exclusively among higher risk African Americans. With the exception of blood pressure, our results are consistent with other studies and contribute to the literature by reporting on the positive effects of a comprehensive lifestyle intervention among African Americans.

### Limitations

There are a few caveats that must be acknowledged in the context of these findings. Highly motivated volunteers are typically recruited in randomized, controlled trials, and thus such participants may be less representatives of the general public. Additionally, our findings were based on African Americans residing in urban Atlanta GA. As a result, generalizability of our findings to the broader African American population may be reduced. However, we used random assignment which leads to high internal validity, particularly when follow-up rates are high and strong quality control procedures are in place. Consequently, observed differences among treatment group probably reflect the effects of the intervention rather than other potentially unmeasured confounding factors. It is anticipated that similar outcome findings would be observed among African Americans in similar areas of the United States.

## 5. CONCLUSION

The results of our study reveal that a short-term comprehensive intervention designed to improve multiple CVD lifestyle risk factors has the potential to improve risk factor profile among higher risk African Americans. They further foster the recommendation that caregivers should advice such patients to engage in a program that offers a comprehensive risk reduction program. Furthermore, public health policymakers should allocate funds to community-based health oriented organizations to implement such programs.

## Figures and Tables

**Table 1:** Baseline characteristics by randomized group^a^.

Characteristic	Treatment n = 68	Control n = 68	*P* value
Age, y	50.2 ± 10.4	47.1 ± 12.5	0.2
Women, %	66.1	60.7	0.6
Education, y^b^	14.7 ± 3.1	15.3 ± 2.3	0.2
SBP, mmHg^c^	140.1 ± 11.1	139.5 ± 15.8	0.8
DBP, mmHg^d^	90.9 ± 9.8	88.2 ± 9.3	0.1
Antihypertensive medication, %	39.3	39.3	1.0
Waist circumference, cm	106.3 ± 16.4	104.6 ± 12.9	0.6
BMI, kg/m^2^^e^	33.6 ± 6.3	34.2 ± 6.1	0.7
Exercise, t/wk^f^	1.2 ± 1.57	2.5 ± 2.0	<0.0001
Fruits/vegetables, Servings/d^g^	1.7 ± 0.9	2.6 ± 1.4	0.002
Level of daily stress	3.2 ± 0.9	3.1 ± 0.9	0.32

a Values represent mean ± SD (standard deviation) for continuous variables and % for categorical variables. *P* values are based on two-sample *t*-test and x^2^, respectively. Level of significance was established as *P* < 0.05.

b education, y = years of education.

c SBP = systolic blood pressure.

d DBP = diastolic blood pressure.

e BMI = body mass index.

f t/wk = times weekly.

g servings/d = servings daily.

**Table 2:** Mean change in outcome variables within and between randomized groups from baseline to 6 month post-intervention follow-up^a^.

Outcome Variable	Within-Group			Pairwise Comparison		

	Treatment Mean ± SD n = 56	*P* value*	Control Mean ± SD n = 56	*P* value	Mean Net Difference Treatment vs Control^b^ (95% CI)^d^	*P* Value
Waist circumference, cm						
Baseline	106.3 ± 16.4		104.6 ± 12.8			
Follow-up	104.1 ± 16.1		108.8 ± 17.7			
Change	−2.2 ± 5.2	0.45	4.2 ± 9.5	0.15	−6.4 (−1.5, 7.5)	<0.001
BMI, kg/m^2^^d^						
Baseline	33.6 ± 6.4		34.4 ± 6.3			
Follow-up	33.4 ± 6.3		34.7 ± 6.8			
Change	−0.2 ± 1.5	0.86	0.3 ± 2.5	0.76	−0.5 (0.01, 1.5)	0.04
Exercise, t/wk^e^						
Baseline	1.6 ± 1.6		2.4 ± 2.0			
Follow-up	3.9 ± 1.3		2.8 ± 1.9			
Change	2.3 ± 1.3	<0.001	0.4 ± 2.1	0.23	1.9 (1.5, 0.3)	0.02
Fruit/vegetable, servings/d^f^						
Baseline	1.7 ± 0.9		2.6 ± 1.4			
Follow-up	3.5 ± 1.4		2.7 ± 1.8			
Change	1.8 ± 1.3	<0.001	0.2 ± 1.9	0.54	1.6 (1.5, 0.4)	0.002
Level of daily stress						
Baseline	3.2 ± 0.9		3.1 ± 0.9			
Follow-up	2.9 ± 0.9		3.2 ± 0.8			
Change	−0.3 ± 0.8	0.46	0.1 ± 0.8	0.88	−0.4 (−0.1, 0.04)	0.3

a Baseline, follow-up and change values are reported as mean (± standard deviation [SD]). Mean net difference is reported as mean (95% CI confidence interval [CI]). *P* values are derived from separate regressions adjusted for age, education in years, gender, and baseline values for primary outcome values based on a two-tailed significance of *P* < 0.05.

b Difference in change calculated as treatment minus change in control.

c Change calculated as follow-up minus baseline Within treatment versus control.

d BMI = body mass index.

e t/wk = times weekly.

f servings/d =servings daily.

**Table 3:** Mean change in blood pressure stratified by overall, medicated, and non-mediated hypertensive respondents within and between randomized groups from baseline to 6 month follow-up^a^.

Outcome Variable	Within-Group			Pairwise Comparison		

	Treatment Mean ± SD	*P* value	Control Mean ± SD	*P* value	Mean Net Difference Treatment vs Control^b^ (95% CI)^d^	*P* Value
				Overall		
	n = 56		n = 56			
SBP, mmHg^c^						
Baseline	140.1 ± 11.1		139.5 ± 15.8			
Follow-up	136.6 ± 12.2		136.1 ± 18.7			
Change^d^	−3.5 ± 13.8	0.39	−3.4 ± 14.8	0.27	−0.1 (−5.0 – 5.2)	0.99
DBP, mmHg^c^						
Baseline	90.9 ± 9.8		88.2 ± 9.3			
Follow-up	83.1 ± 8.4		84.8 ± 11.0			
Change	−7.8 ± 9.8	0.002	−3.4 ± 11.0	0.07	−4.4 (−5.5 - 1.4)	0.90
				Medicated		
	n = 22		n = 22			
SBP, mmHg						
Baseline	143.0 ± 11.7		141.3 ± 14.9			
Follow-up	137.1 ± 14.2		139.2 ± 17.7			
Change	−5.9 ± 17.5	0.39	−2.1 ± 13.6	0.68	−3.8 (−12.1 - 5.3)	0.98
DBP, mmHg						
Baseline	0.07		86.0 ± 10.5			
Follow-up	0.07		83.7 ± 11.6			
Change	0.07	0.07	−2.3 ± 9.87	0.18	−3.54 (−9.3 - 2.8)	0.60
				Non-medicated		
	n = 34		n = 34			
SBP, mmHg						
Baseline	138.2 ± 10.5		138.4 ± 14.9			
Follow-up	135.6 ± 11.2		133.1 ± 19.4			
Change	−2.6 ± 11.0	0.35	−5.3 ± 15.7	0.37	−2.7 (−4.8 – 10.0)	0.60
DBP, mmHg						
Baseline	92.4 ± 8.0		89.6 ± 8.3			
Follow-up	87.3 ± 7.1		85.9 ± 10.5			
Change	−5.1 ± 9.1	0.07	−3.7 ± 11.1	0.45	−1.4 (−6.3 - 3.4)	0.80

a Baseline, follow-up and change values are reported as mean (±SD). Mean net difference is reported as mean (95% confidence interval [CI]). *P* values are derived from separate regressions adjusted for age, education in years, gender, and baseline values for primary outcome variables based on a two-tailed significane of *P* < 0.05.

b Difference in change calculated as treatment minus change in control.

c SBP = systolic blood pressure; DBP = diastolic blood pressure.

d Change calculated as follow-up minus baseline within treatment versus control group.
